# Case Report: ECMO-assisted tracheal reconstruction in a 30-week-gestation preterm infant with tracheal stenosis

**DOI:** 10.3389/fped.2024.1363419

**Published:** 2024-03-04

**Authors:** Zi-Hao Li, Shi-Hao Li, Zhen-Yang Geng, Bin Wu, Yin-Liang Sheng, Ping Yuan, Feng Li, Yu Qi

**Affiliations:** Department of Thoracic Surgery, First Affiliated Hospital of Zhengzhou University, Zhengzhou, China

**Keywords:** tracheal stenosis, premature infant, tracheal reconstruction, ECMO, case report

## Abstract

Tracheal stenosis is a rare but life-threatening disease in preterm infants. Misdiagnosis as congenital tracheal stenosis is common, making surgical management challenging. This report presents a case of a preterm infant with tracheal stenosis and congenital heart malformation treated with ECMO-assisted tracheal resection and end-to-end anastomosis. A male infant was born at 30 weeks of gestation with severe asphyxia, cardiac insufficiency, and pneumonia. Following failed medical treatment, fiberoptic bronchoscopy confirmed mid-tracheal to carinal stenosis. After a 2-week treatment course, ECMO-assisted tracheal resection and end-to-end anastomosis were performed successfully. This case confirms the feasibility of tracheal resection and end-to-end anastomosis in low-weight, preterm infants with tracheal stenosis born at 30 weeks gestation. The utilization of ECMO for oxygenation during surgery provides a clear surgical field and shorter operating time. Surgical intervention may be necessary for neonatal tracheal stenosis depending on the clinical presentation.

## 1 Introduction

In preterm infants, tracheal stenosis is an uncommon but challenging disease. The condition is often misdiagnosed as congenital tracheal stenosis(CTS) which is characterized by intact cartilage rings of the trachea (1). Tracheal stenosis in preterm infants is life-threatening and multiple complications accompanying (2). Furthermore, preterm infants have peculiar characteristics such as a smaller airway, increased oxygenation requirements, and greater ductility of the tracheal cartilage wall, which could result in greater anastomotic difficulty and limitation of airflow postoperatively (3). Therefore, surgical management of preterm infants with tracheal stenosis is challenging, and the difficulty of surgery is often exacerbated by other comorbid congenital conditions (2).

In this report, we describe a case of a preterm infant at 30 weeks of gestation with tracheal stenosis and congenital heart malformation, which led to severe asphyxia at birth, cardiac insufficiency, and pneumonia. We implemented an ECMO-assisted tracheal resection and end-to-end anastomosis in this infant, which confirmed the feasibility of tracheal resection and end-to-end anastomosis in preterm infants at 30 weeks gestation.

## 2 Case description

### 2.1 Patient

A male infant was born at 30 weeks of gestation. As a result of severe preeclampsia, placenta previa, abruptio placenta, amniotic fluid contamination, and intrauterine distress, his mother had a cesarean section at a local hospital. The infant's neck was wrapped around by the umbilical cord for 2 weeks before birth and he weighed only 1,380 g at birth. The APGAR scores were 2 after 1 min (1 point each for heart rate and respiration). After tracheal intubation and resuscitation balloon pressor oxygen rescue, the APGAR scores were 3 at 5 min (1 point each for heart rate, reflex, and muscle tone) and 4 at 10 min (1 point each for heart rate, respiration, reflex, and skin color). He was admitted to the NICU of the local hospital where the infant was given invasive ventilator-assisted ventilation and anti-infective therapy (with merocillin, cefoperazone sulbactam, and penicillin). He was also treated with two sequential supplements of PS (bovine pulmonary surfactant 140 mg and porcine pulmonary surfactant 240 mg) as well as a transfusion of component blood (plasma, red blood cells) to improve coagulation and correct anemia. Following 8 days of treatment, no significant effects were seen, and 1 day later, the infant's dyspnea worsened suddenly with sobbing-like breathing. His family sought additional diagnosis and treatment, and they contacted our hospital's emergency transport vehicle. The infant was transported to our hospital with medical and nursing staff from our hospital, while undergoing endotracheal intubation and assisted ventilation.

After admission, we provided specialized and intensive care for the neonate, who was placed within a warm incubator with assisted ventilation. Concurrently, we administered various therapies including antibiotics in combination with anti-infective agents, cardiomyocyte nourishment, hemorrhage prevention, cardiovascular circulation improvement, pulmonary hypertension reduction, cardiac function enhancement, multivitamin, energy, and trace element supplementation. The neonate underwent two consecutive failed attempts of ventilator withdrawal. Subsequent fiberoptic bronchoscopy disclosed a progressive mid-tracheal to carinal stenosis, with a significant reduction in diameter approximately 0.5 cm above the carina. The most constricted segment allowed for a mere 2.5 mm outer diameter bronchoscope to pass through. Following this, the carina became visible, but the right main bronchus was obstructed by secretions, which we relieved post-irrigation with normal saline. The left main bronchus appeared comparably unobstructed. Ultimately, we administered diluted epinephrine-saline spray into the lower tracheal stenosis. Chest radiography demonstrated bilateral pulmonary inflammation, while CTA indicated atrial septal defects, decreased lung lucency bilaterally, and bilateral pulmonary inflammation. There was local narrowing above the tracheal carina, as well as in the bronchial localization of the right middle segment. Subsequently, based on initial assessments, possible diagnoses included tracheal stenosis, respiratory failure, atrial septal defects, cardiac insufficiency, severe birth asphyxia, neonatal infection, neonatal anemia, neonatal hyperglycemia, neonatal hypocalcemia, neonatal hypoproteinemia, prematurity, and very low birth weight.

**Figure 1:**
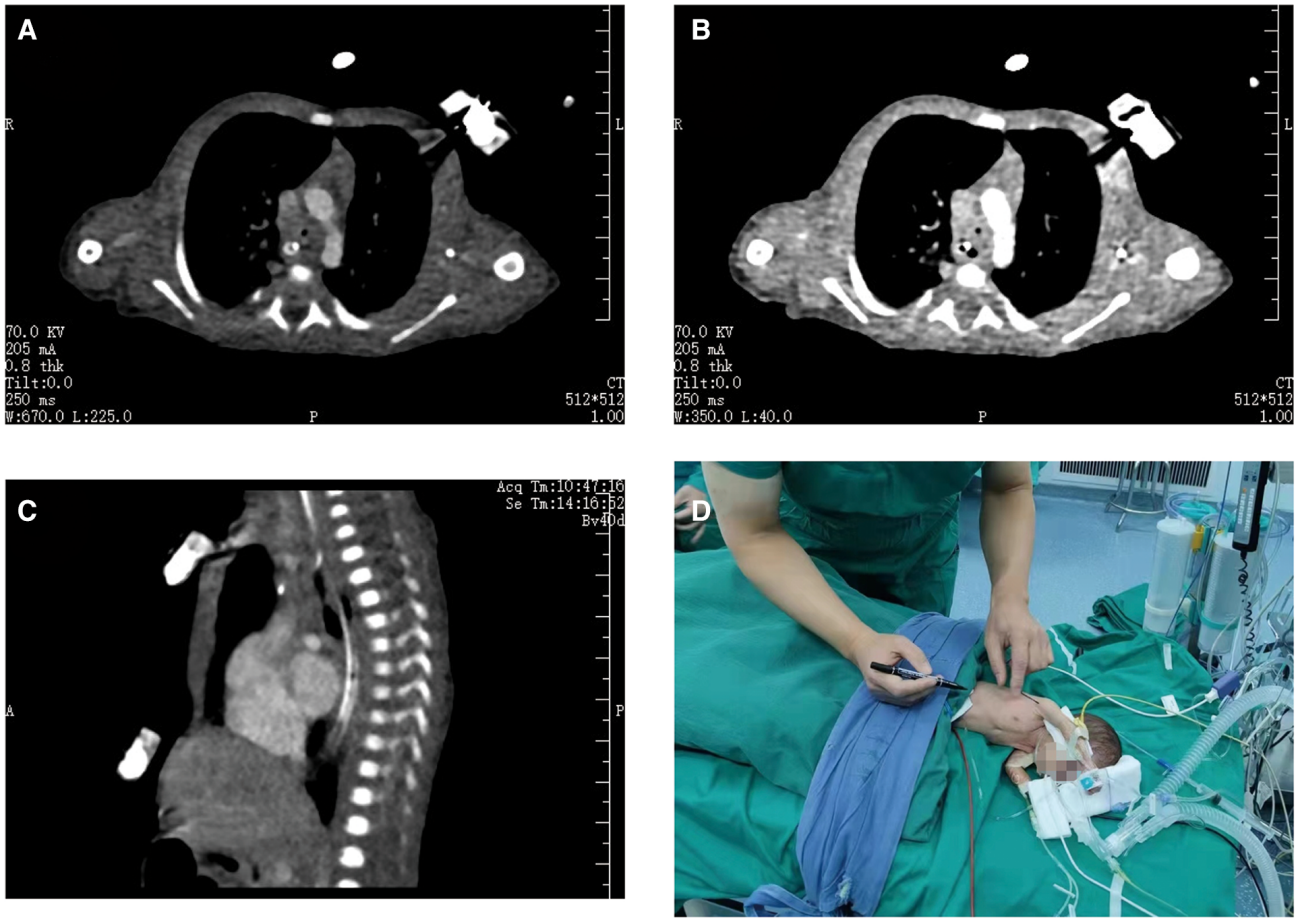
Preoperative examination of the patient. A computed tomography scan of his chest revealed that the lumen above the tracheal carina was narrow, with a diameter of approximately 1.7 mm at its narrowest point and involvement of approximately 7 mm in length (**A**–**C**). We were going to operate on the infant with ECMO-assisted tracheal resection and end-to-end anastomosis (**D**).

After a 2-week course of treatment at our hospital, the infant's weight was recorded at 1.47 kg, with normalization of infection indicators and improvement in CSF brightness and blood biochemical indicators. The infant exhibited slightly increased respiration, weakly positive three concave signs, and no apparent cardiac auscultation abnormalities. A chest computed tomography scan showed stenosis above the tracheal carina, with a narrowest diameter of approximately 1.7 mm and a length of approximately 7 mm (Figures 1A–C). To address the issue of tracheal stenosis in the infant, we decided to perform an ECMO-assisted tracheal resection and end-to-end anastomosis on the 22nd day after birth, following exclusion of surgical contraindications (Figure 1D). Informed consent was obtained from the families.

### 2.2 Surgical procedure

**Figure 2:**
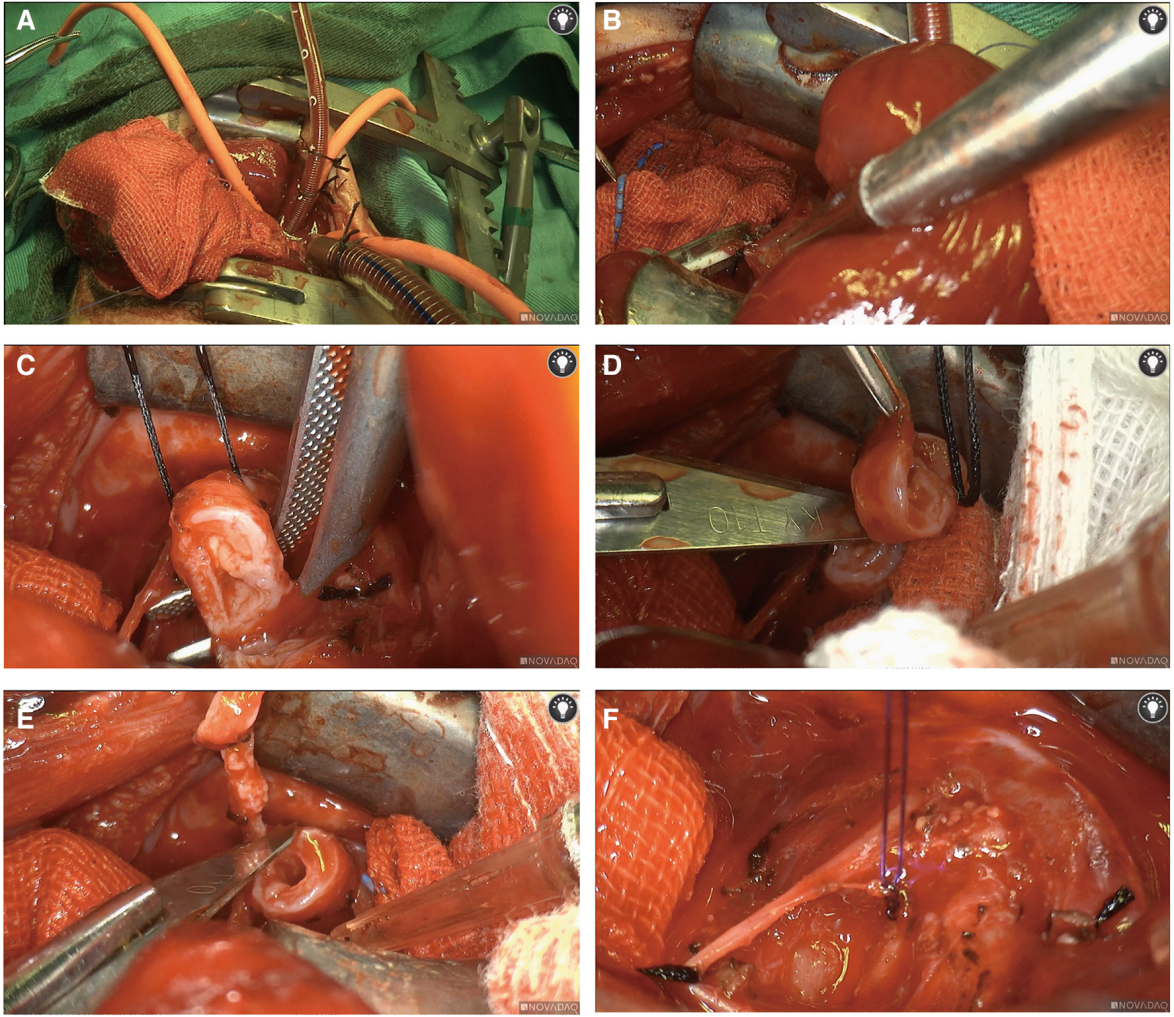
Surgical procedures. Central venoarterial (VA) ECMO with right atrial cannulation for venous drainage and aortic return cannulation was used (**A**) We isolated the azygos vein and doubly ligated and divided the azygos vein with a 3-0 suture (**B**) In a position anterior to the trachea and posterior to the phrenic nerve, we opened the mediastinal pleura, freeing out the lower trachea. The distal and proximal segments of the tracheal stenosis were cut with a scalpel, and the tracheal stenosis segment was removed, followed by continuous suturing of the tracheal stump with 4-0 absorbable sutures to complete an end-to-end anastomosis (**C**–**F**).

Following intravenous administration of general anesthesia, the neonate was positioned in left lateral decubitus for routine disinfection and draping. An incision of approximately 8 cm was made at the 4th intercostal space in the right axilla. Upon entering the thoracic cavity, we established ECMO access using central venoarterial (VA) cannulation with right atrial drainage and aortic return cannulation, connected to 1/4” inner diameter tubes (Figure 2A). The ECMO circuit consisted of a centrifugal pump with a membrane oxygenator (LILLIPUT 2 ECMO), and heparin was used to maintain an activated clotting time (ACT) of around 350 s.

We then isolated the azygos vein and divided it after doubly ligating it with a 3-0 suture (Figure 2B). After freeing the lower trachea by opening the mediastinal pleura, we resected approximately 8 mm of the tracheal segment with a scalpel in a position anterior to the trachea and posterior to the phrenic nerve. We completed the end-to-end anastomosis by suturing the distal and proximal segments of the tracheal stump continuously with 4-0 absorbable sutures (Figures 2C–E). Thoracic irrigation was performed, and lung inflation was observed without bubble spillage, indicating successful anastomosis and adequate lung inflation.

**Figure 3:**
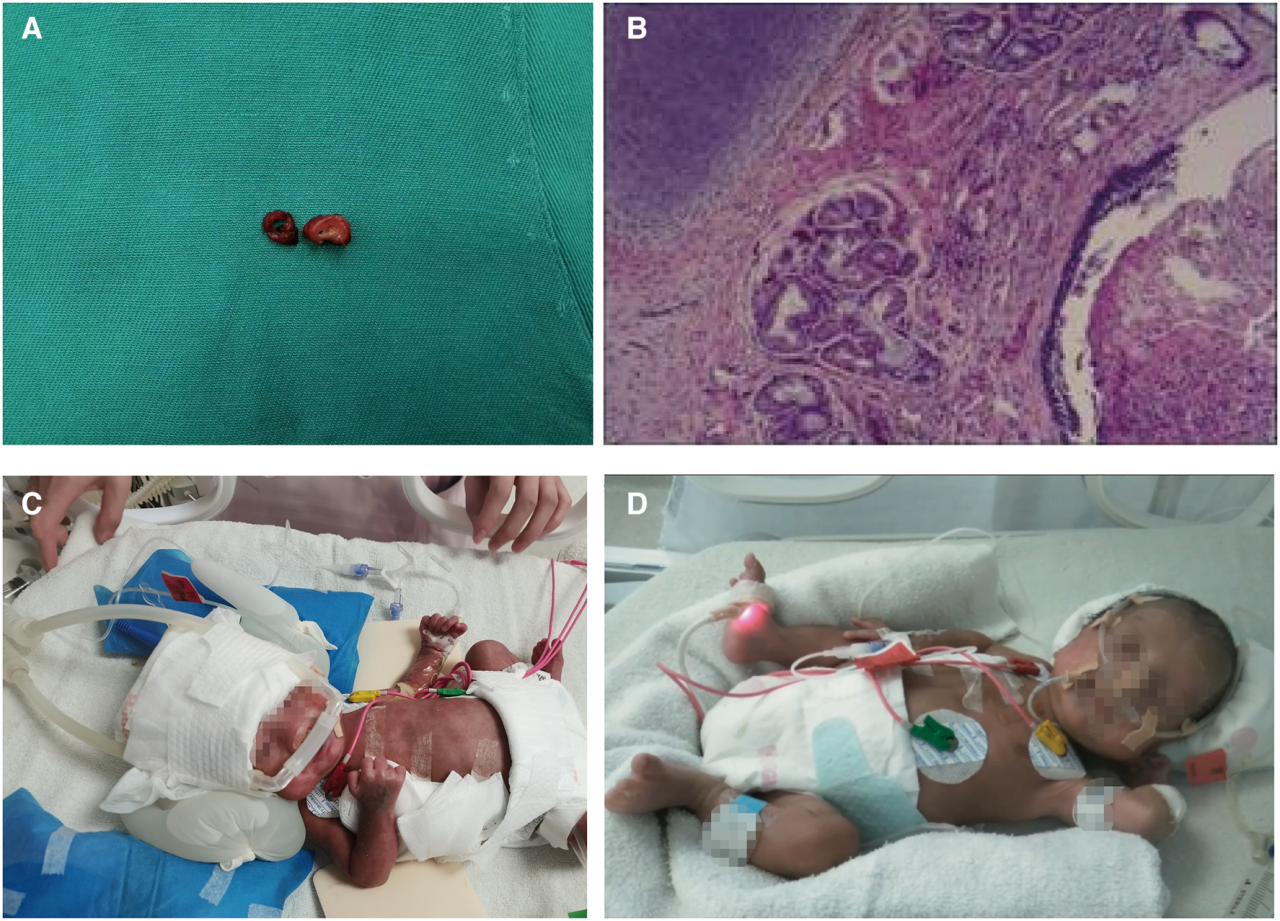
Postoperative examination of the patient. (**A**) Tracheal stenosis segment and tracheal margins; (**B**) Postoperative pathological results revealed chronic inflammation of bronchial mucosa with polypoid hyperplasia. On the fourth postoperative day, the infant's endotracheal tube was removed and switched to nasal noninvasive ventilator-assisted ventilation (**C**) After two chest drains were removed, the infant continued to receive treatment in a warm box (**D**).

Hemostatic material was applied to the wound due to its large size, but no suture fixation was performed on the preterm infant's head due to the short length of the resected tracheal segment. The ECMO was withdrawn after 83 min of use, and two closed thoracic drainage tubes were placed. The chest was closed layer by layer after confirming instrumentation inventory without error. The infant was then transferred to the NICU for postoperative tracheal intubation, and specimens were sent to the pathology department while also being presented to the family (Figure 3A).

## 3 Results

Postoperatively, the infant was administered dexamethasone to reduce laryngeal edema and was treated with a combination of meropenem and vancomycin for anti-infective therapy. Inhaled budesonide was also administered to attenuate airway inflammation, and systemic intravenous nutrition was given. Postoperative pathological results indicated chronic inflammation of the bronchial mucosa with polypoid hyperplasia (Figure 3B). The two segments of tracheal tissue sent for examination were 5 mm and 3 mm wide, respectively, and no complete tracheal rings were seen. On the fourth postoperative day, the infant's endotracheal tube was replaced with nasal noninvasive ventilator-assisted ventilation (Figure 3C), and the two chest drains were removed on postoperative Day 10.

**Figure 4:**
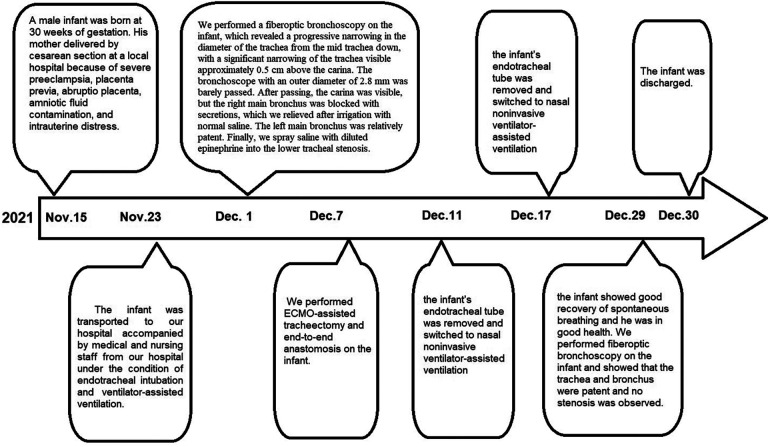
A timeline for the entire process with relevant data from the episode of care.

The infant continued treatment in an incubator while requiring oxygen supply-assisted breathing, but gradually recovered, showing a negative three concave sign. Twenty-four days postoperative, fiberoptic bronchoscopy revealed patent trachea and bronchi without stenosis. The infant was discharged without complications, and the family expressed gratitude for the successful treatment. Now is one year later, and the child has recovered with a weight gain of 13 kg. A timeline with relevant data from the episode of care is presented in Figure 4.

## 4 Discussion

Tracheal stenosis occurring in preterm infants is a rare, but potentially life-threatening disease that may be misdiagnosed as congenital tracheal stenosis. The disparity between neonatal tracheal stenosis and congenital tracheal stenosis in newborns stems from their distinct etiologies. Congenital tracheal stenosis in newborns is triggered by inherent structural abnormalities, whereas neonatal tracheal stenosis results from postnatal factors like inflammation, infection, or trauma. Moreover, while congenital tracheal stenosis in newborns can typically be diagnosed shortly after birth, it may take longer to diagnose neonatal tracheal stenosis (4). Treatment modalities also diverge and mandate an individualized therapeutic approach tailored to the underlying etiology and severity of each case. Surgical intervention is frequently required for congenital tracheal stenosis, while medical management or surgical intervention may be indicated for neonatal tracheal stenosis depending on the specific clinical presentation (4, 5). Considering the age of the newborn and the urgency of the situation, we prioritized a more immediate and comprehensive approach, which led us to the decision to proceed with emergency tracheal reconstruction surgery. Given the critical nature of the condition, we believed that surgical intervention offered a more rapid and decisive resolution to the acute airway compromise. While balloon dilation can be a viable option in certain cases of tracheal stenosis, our decision in this instance was driven by the specific circumstances, the severity of the condition, and the need for urgent intervention to ensure the best possible outcome for the newborn.

Tracheal surgery in infants and young children is a complex procedure that carries a significant risk of morbidity and mortality, particularly in patients with cardiac anomalies. When not involving the heart, our preferred method of extracorporeal life support is ECMO. Therefore, we have implemented an innovative approach of utilizing ECMO for oxygenation as opposed to endotracheal intubation, resulting in clearer surgical field and shorter operating time (6). Over the past two years, we have performed multiple successful ECMO-assisted tracheal tumor resections with tracheal reconstruction, thereby accumulating extensive experience in this field (7). As a result, we continue to use ECMO as our primary approach to ensure optimal surgical outcomes. Given the delicate nature of infant vasculature, we employed central venoarterial (VA) ECMO with right atrial cannulation for venous drainage and aortic return cannulation.

Our case is unique in that the infant's poor general condition at birth, prematurity at 30 weeks gestation with a birth weight of only 1,380 g, and reliance on endotracheal intubation for assisted breathing. Additionally, the patient had a congenital atrial septal defect, and severe pulmonary infection. Following symptomatic treatment, we were able to control the infection and perform a successful surgery on this high-risk, preterm infant. The surgical procedure, which involved removing a stenotic segment of the trachea with anastomosis, was completed in under two hours, reducing postoperative recovery time. This confirms the feasibility of such a procedure in low-weight, preterm infants with tracheal stenosis born at 30 weeks gestation.

In conclusion, while the incidence of tracheal stenosis in infants is relatively low, it is imperative to choose appropriate surgical modalities as early as possible in order to save more lives. The ECMO-assisted tracheal resection and end-to-end anastomosis procedure that we performed on this infant may serve as a valuable reference for our peers in their efforts to treat similar patients.

## Data Availability

The raw data supporting the conclusions of this article will be made available by the authors, without undue reservation.

## Supplementary material

SUPPLEMENTARY VIDEO S1

Main surgical procedure.

## References

[1] Hewitt RJ Butler CR Maughan EF Elliott MJ. Congenital tracheobronchial stenosis. Semin Pediatr Surg. (2016) 25(3):144–9. 10.1053/j.sempedsurg.2016.02.007 27301600

[2] Hoetzenecker K Schweiger T Denk-Linnert DM Klepetko W. Pediatric airway surgery. J Thorac Dis. (2017) 9(6):1663–71. 10.21037/jtd.2017.05.50 28740684 PMC5506117

[3] Hoetzenecker K Klepetko W Keshavjee S Cypel M. Extracorporeal support in airway surgery. J Thorac Dis. (2017) 9(7):2108–17. 10.21037/jtd.2017.06.17 28840012 PMC5542968

[4] Hofferberth SC Watters K Rahbar R Fynn-Thompson F. Management of congenital tracheal stenosis. Pediatrics. (2015) 136(3):e660–9. 10.1542/peds.2014-3931 26304826

[5] Wen W Du X Zhu L Wang S Xu Z Lu Z. Surgical management of long-segment congenital tracheal stenosis with tracheobronchial malacia. Eur J Cardiothorac Surg. (2022) 61(5):1001–10. 10.1093/ejcts/ezab551 34940823

[6] Pola Dos Reis F Minamoto H Bibas BJ Minamoto FEN Cardoso PFG Caneo LF Treatment of tracheal stenosis with extracorporeal membrane oxygenation support in infants and newborns. Artif Organs. (2021) 45(7):748–53. 10.1111/aor.13898 33350476

[7] Li ZH Dong B Wu CL Li SH Wu B Sheng YL Case report: ECMO-assisted uniportal thoracoscopic tracheal tumor resection and tracheoplasty: a new breakthrough method. Front Surg. (2022) 9:859432. 10.3389/fsurg.2022.859432 35445074 PMC9013741

